# Post-operative Outcomes Following Urgent and Elective Surgery for Ulcerative Colitis: A Systematic Review and Meta-Analysis

**DOI:** 10.7759/cureus.97100

**Published:** 2025-11-17

**Authors:** Shuvam Sarkar, Revathy Latheesh

**Affiliations:** 1 General Surgery, Queen Elizabeth University Hospital, Glasgow, GBR; 2 General Surgery, University of Dundee, Dundee, GBR

**Keywords:** elective surgeries, emergent colectomy, perioperative outcomes, ulcerative colıtıs, urgent surgeries

## Abstract

Ulcerative colitis (UC) is a chronic inflammatory condition affecting the large bowel. Surgery remains an important cornerstone in the treatment framework for UC, both in the elective setting for patients refractory to medical management and in the urgent setting for patients with acute severe UC. The aim of this study is to better define the risks associated with urgent surgery for acute severe UC when compared with elective surgery. This would allow clinicians to better outline the risks associated with urgent and elective bowel resections for UC and enable patients to make more informed decisions.

A systematic review of the PubMed database was conducted independently by two authors looking at post-operative outcomes following bowel resection for UC within the elective and urgent settings. The review was conducted in accordance with the Preferred Reporting Items for Systematic Reviews and Meta-Analyses (PRISMA) guidelines. Inclusion and exclusion criteria were pre-established using the Population, Intervention, Comparison, Outcome and Study (PICOS) framework. The primary outcome was 30-day mortality post-operatively and secondary outcomes were infection and re-operation rates following elective and urgent surgery. Bias was assessed using the Newcastle-Ottawa Score. A random effects meta-analysis was carried out. The absolute risk difference was calculated with 95% confidence intervals and p values for each of the primary and secondary outcomes. The systematic review yielded nine studies which were ultimately included within the meta-analysis. Infection rates following operation were included in two of these studies. Re-operation rates were included in four of these studies. A total of 5797 patients underwent urgent surgery and 12479 patients underwent elective surgery across the nine articles. Eight studies had a low risk of bias and one study had a medium risk of bias when assessed against the Newcastle-Ottawa Score. Overall, there was a 4.8% increased risk of mortality at 30 days post-operatively following urgent surgery when compared to elective surgery (Risk Difference (RD) = 0.048, 95% CI [0.027; 0.069], p < 0.001). This was statistically significant but the data showed significant heterogeneity (Q = 66.6, I^2^ = 88%). There was a 13% increased risk of post-operative infection with urgent surgery compared to elective surgery; however, this was not statistically significant (RD = 0.13, 95% CI [-0.01; 0.27], p = 0.20). There was a 4.5% increased risk of re-operation with urgent surgery; however, this was not statistically significant (RD = 0.045, 95% CI [-0.018; 0.109], p = 0.48).

Patients undergoing urgent bowel resection surgery for UC therefore face a greater risk of mortality at 30 days post-operatively compared to elective surgery. This may be explained by patients in the acute setting being more de-conditioned and co-morbid than their elective counterparts. There was no statistically significant difference in rates of post-operative infection and return to the operating theatre following urgent versus elective surgery for UC. This could be due to better medical treatments allowing for better pre-operative planning and involvement of the multi-disciplinary team in the acute setting. Ultimately, the results from this study provide a reference frame for clinicians when managing patients with UC.

## Introduction and background

Ulcerative colitis (UC) is a chronic inflammatory condition that primarily affects the mucosal and submucosal layers of the large intestine [1]. UC is the most prevalent form of inflammatory bowel disease, with epidemiological evidence suggesting one in every thousand people in the Western hemisphere is affected [2].

Whilst the advent of novel biologics has led to more patients with medically controlled inflammatory bowel disease, surgery remains integral to the treatment framework for UC [3]. Surgery for UC can be undertaken in an elective or emergent setting [4]. The main indications for urgent surgery in the context of acute severe UC include: disease refractory to medical management, uncontrollable sepsis, colonic perforation, toxic megacolon, and severe bleeding [5]. This is generally done as a lifesaving intervention. On the other hand, elective surgery is often undertaken with greater planning, involving the wider multi-disciplinary team and with patients in a more optimised physiological state. Indications for elective surgery in UC include medically refractory colitis, dysplasia, and cancer [5].

Studies have established several pre-operative risk factors that can negatively impact post-operative outcome. This includes haemodynamic instability, co-morbidities, low serum albumin levels, increased age, poor nutritional status, raised inflammatory markers, long-term steroid and immunosuppressant use [6,7]. In an emergent circumstance, many of these factors cannot be adequately controlled, therefore resulting in unfavourable outcomes and increasing post-operative morbidity and mortality [7]. Whilst several studies have investigated post-operative outcomes with UC, there remains a gap in established evidence comparing elective and urgent surgery to determine how outcomes might differ.

The aim of this study is to compare the mortality and morbidity following urgent surgery for acute severe UC with elective surgery. In the acute setting, this would allow clinicians to better outline the risks associated with urgent bowel resections for UC. Electively, this would enable patients to make more informed decisions by contextualising the risk of elective surgery for UC against further possible acute attacks and subsequent emergency operations.

## Review

Methods

Study Protocol

This systematic review protocol was clearly established and submitted prospectively to the PROSPERO database, an international register for systematic reviews, and is available at: https://www.crd.york.ac.uk/PROSPERO/view/CRD420251132276. The review was conducted in accordance with the Preferred Reporting Items for Systematic Reviews and Meta-Analyses (PRISMA) guidelines.

Inclusion and Exclusion Criteria

Inclusion and exclusion criteria were determined with the Population, Intervention, Comparison, Outcome and Study (PICOS) framework. All randomised control trials, cohort studies and case-controlled studies published in English within the last 20 years were included. Notably, there was some heterogeneity in how urgent surgery was defined by different studies. Inclusion was therefore dependent on clear distinction of data between elective and non-elective settings. Any studies including children or combining UC with Crohn's disease were excluded (Table 1).

**Table 1:** Inclusion and exclusion criteria for the systematic review using the Population, Intervention, Comparison, Outcome and Study (PICOS) framework.

	Inclusion	Exclusion
Population	Adults (Age 18 or over) with Ulcerative Colitis	Children (Anyone aged less than 18), Patients with Crohn's Disease or an alternative diagnosis other than ulcerative colitis
Intervention	Surgery with bowel resection within an urgent/emergency setting	Surgery without resection of bowel (e.g. De-functioning stoma) or surgery unrelated to ulcerative colitis in patients with ulcerative colitis (e.g. Cholecystectomy in a patient with ulcerative colitis)
Comparison	Surgery with bowel resection in an elective setting	Surgery without resection of bowel (e.g. Stoma reversal) or surgery unrelated to ulcerative colitis in patients with ulcerative colitis (e.g. Cholecystectomy in a patient with ulcerative colitis)
Outcome	Post-operative 30-day mortality is primary outcome. Secondary outcomes are post-operative infection and re-operation.	Studies not including mortality rates or post-operative outcomes
Study	Randomised Controlled Trials, Cohort studies and Case-Control studies written in English and published in last 20 years	Studies published in languages other than English and studies published more than 20 years ago

The primary outcome was post-operative mortality at 30 days. Secondary outcomes were post-operative infection and re-operation. Post-operative infection was classified as any reported infection that was at least Grade II on the Clavien-Dindo Scale [8]. This ensured inclusion of deep-seated wound infections as well as intra-abdominal infections such as collections and anastomotic leaks. Where only superficial wound infection rates were reported, this was excluded from the post-operative infection data set.

Systematic Literature Review

A systematic literature search was conducted independently by both authors within the PubMed database. The search code is shown in Appendix 1. Further searches within the reference list of relevant articles were also conducted. Studies were screened using the pre-established inclusion and exclusion criteria. Any disagreements between the authors were settled with discussion in the first instance. Ongoing disagreement regarding study inclusion was planned to be resolved by involving a neutral third party; however this was ultimately not required.

**Figure 1:**
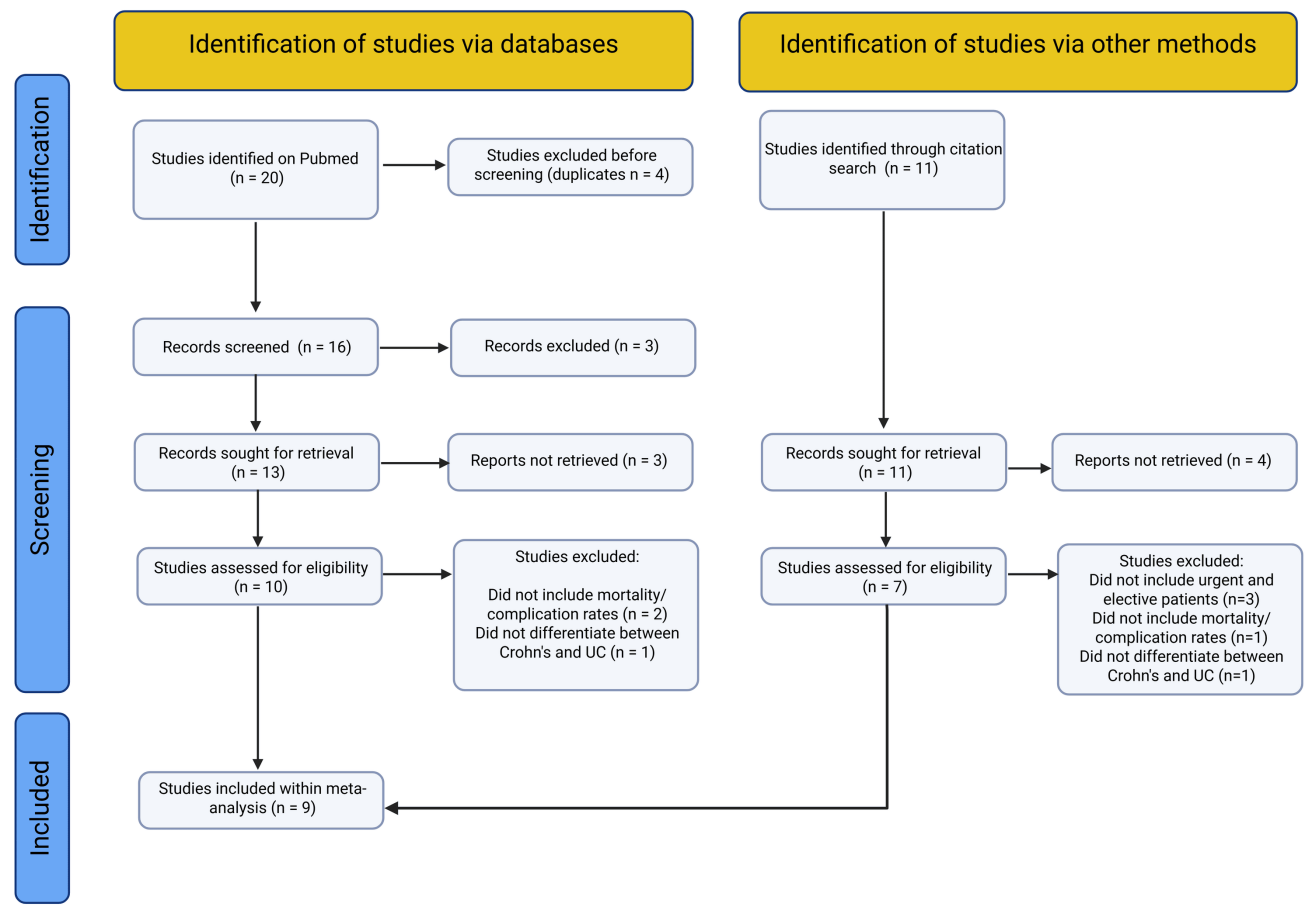
Preferred Reporting Items for Systematic Reviews and Meta-Analyses (PRISMA) Flowchart Shows the screening process for articles within the systematic review in accordance with the Preferred Reporting Items for Systematic Reviews and Meta-Analyses (PRISMA) guidelines [9].

The review process and individual reasons for study exclusion are outlined in the PRISMA flowchart (Figure 1).

Bias

Both authors then independently reviewed the included studies for data on study design and outcomes. The Newcastle-Ottawa Scale was used to assess for bias and each study was scored for Selection (out of four stars), Comparability (out of two stars) and Outcome (out of three stars) [10]. A funnel plot was created and Egger's test was conducted to assess for publication bias.

Meta-Analysis

Due to the heterogeneity between studies, a Der Simonian-Laird random effects (RE) meta-analysis was carried out. The absolute risk difference (RD) was calculated with 95% confidence intervals (CI) and p values for each of the primary and secondary outcomes. To analyse for variance between studies, statistical analysis was done to establish Cochran's Q test, degrees of freedom (df) and I^2^ statistic for each outcome data set. Sensitivity analysis was assessed by omitting each study in turn and repeating the meta-analysis. Subgroup analysis was attempted with BMI and pre-operative ASA grading but raw data was not available within the included studies.

Results

Literature Review

Preliminary search on PubMed yielded 20 studies and a further 11 studies were found through reference list searches. A total of 17 studies underwent full literature review and nine studies were ultimately included within the meta-analysis [11-19]. Infection rates following operation were included in two of these studies [15,16]. Re-operation rates were included in four of these studies [13-15,18]. A total of 5797 patients underwent urgent surgery and 12479 patients underwent elective surgery across the nine articles.

Assessment of Bias

The studies were assessed for bias in accordance with the Newcastle-Ottawa Scale. The Newcastle-Ottawa Score was calculated with maximum of four stars for selection of the study, two stars for comparability and three stars for outcome (Table 2).

**Table 2:** Assessment of Bias using the Newcastle-Ottawa Scale Shows the number of patients outlined by each study, the type of study, which outcomes were listed in each study and the Newcastle-Ottawa Score.

Study	Number Of Patients	Study Type	Outcomes included	Newcastle-Ottawa Scale
Roberts et al. 2007 [11]	1983	Retrospective, Cohort	Mortality	Selection xxxx Comparability xx Outcome xx
Kaplan et al. 2008 [12]	6266	Retrospective, Cohort	Mortality	Selection xxxx Comparability xx Outcome xx
De Silva et al. 2011 [13]	591	Retrospective, Cohort	Mortality, Re-operation	Selection xxxx Comparability xx Outcome xxx
Tottrup et al. 2012 [14]	1994	Retrospective, Cohort	Mortality, Re-operation	Selection xxxx Comparability xx Outcome xxx
Patel et al. 2013 [15]	4962	Retrospective, Cohort	Mortality, Infection, Re-operation	Selection xxxx Comparability xx Outcome xxx
Hicks et al. 2014 [16]	179	Retrospective, Cohort	Mortality, Infection	Selection xxxx Comparability xx Outcome xx
Ikeuchi et al. 2014 [17]	144	Retrospective, Cohort	Mortality	Selection xxx Comparability xx Outcome xx
Burns et al. 2022 [18]	56	Retrospective, Cohort	Mortality, re-operation	Selection xxx Comparability xx Outcome xxx
Couch et al. 2025 [19]	2101	Retrospective, longitudinal cohort	Mortality	Selection xxxx Comparability x Outcome xxx

**Figure 2:**
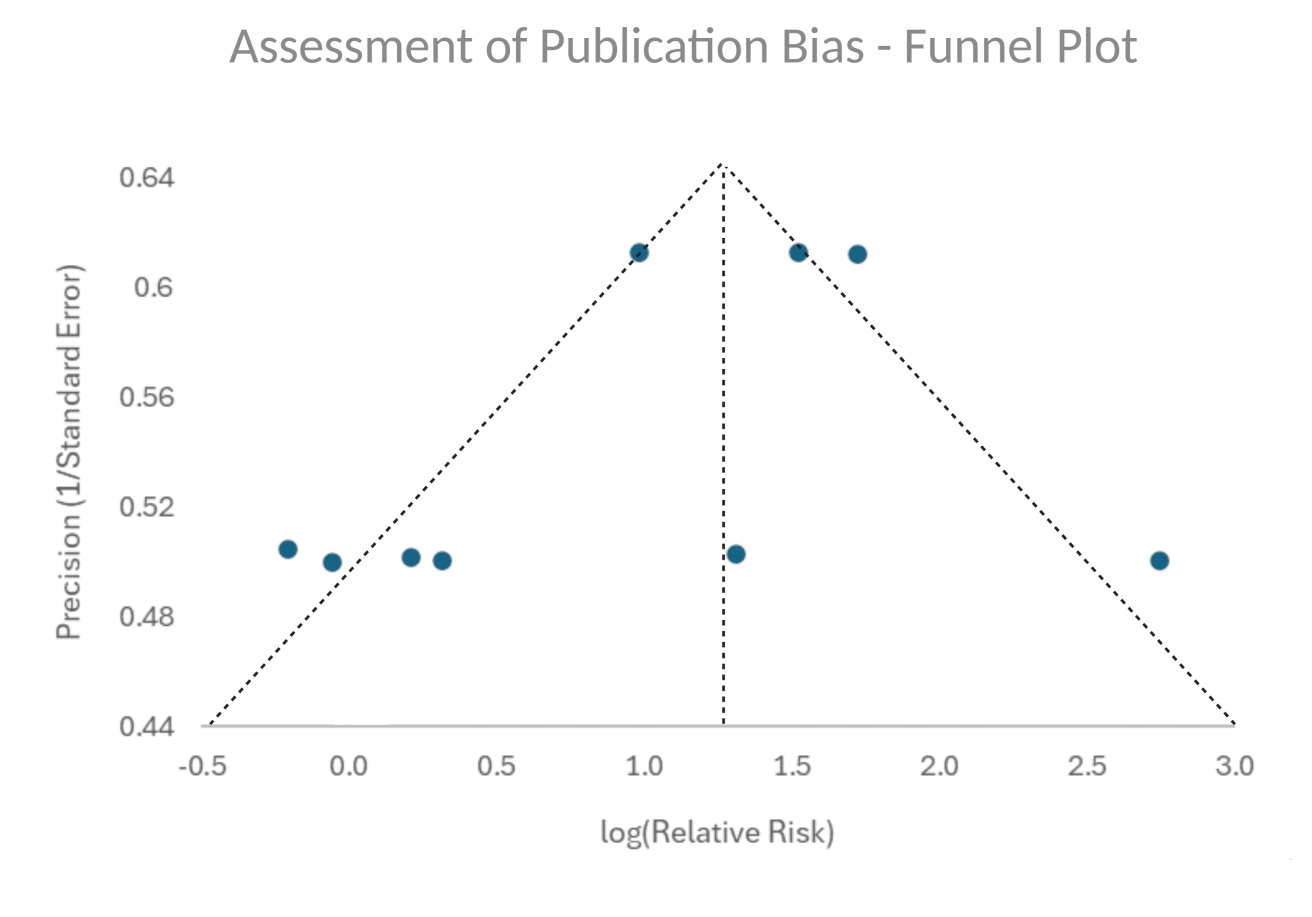
Assessment of Publication Bias - Funnel Plot Funnel plot showing the logarithm of relative risk for mortality (x-axis) against the inverse of standard error (y-axis). Vertical dotted line shows relative risk for mortality from pooled data from all studies. Symmetrical distribution of studies shown.

Publication bias was assessed with a funnel plot, which showed even distribution of studies across the pooled data (Figure 2). Egger's test was also conducted, which showed a p value of 0.31.

Post-operative Mortality

**Figure 3:**
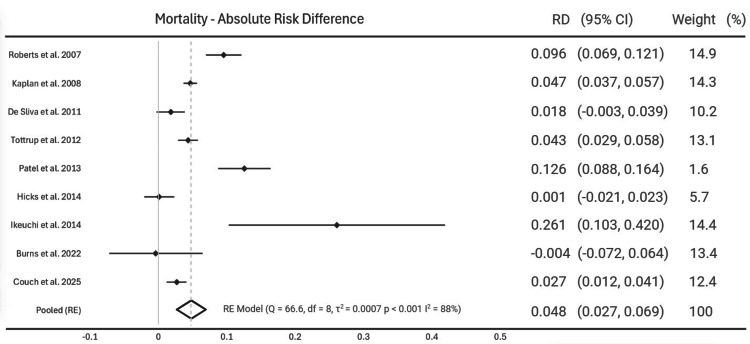
Mortality - Absolute Risk Difference Forest plot showing absolute risk difference in 30-day mortality following urgent surgery compared to elective surgery. Study weights are given for each study on the right hand column [11-19].

Post-operative mortality rates were found in all nine studies, with six of the studies showing a statistically significant increase in mortality following urgent surgery compared to elective surgery. Pooled random effect analysis showed a 0.048 increase in mortality with urgent surgery (95% CI [0.027; 0.069], p < 0.001). Heterogeneity assessment showed a Q test value of 66.6 and I^2^ of 88% (Figure 3).

Post-operative Infection

**Figure 4:**
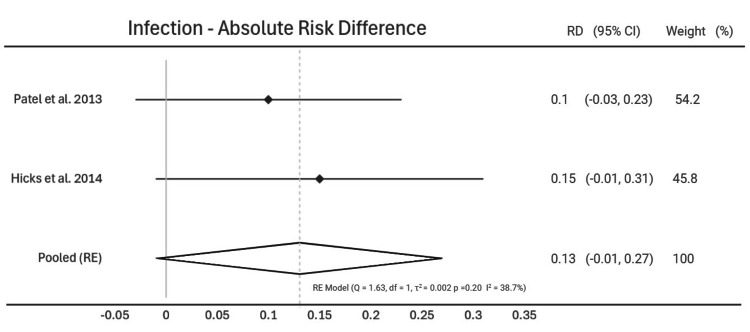
Infection - Absolute Risk Difference Forest plot showing absolute risk difference in post-operative infection rates following urgent surgery compared to elective surgery. Study weights are given for each study on the right hand column [15,16].

Post-operative infection rates were outlined in two of the studies [15,16]. Pooled random effect analysis showed a 0.13 increase in absolute risk of infection with urgent surgery (95% CI [-0.01; 0.27], p = 0.20). Heterogeneity assessment was conducted (Q = 1.63, p = 0.20, I^2^ = 38.7%) (Figure 4).

Re-operation

**Figure 5:**
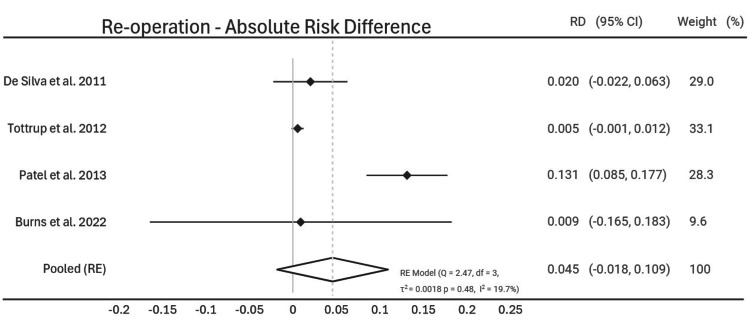
Re-Operation - Absolute Risk Difference Forest plot showing absolute risk difference in re-operation rates following urgent surgery compared to elective surgery. Study weights are given for each study on the right hand column [13-15,18].

The rates for re-operation following elective versus urgent surgery were outlined in four of the studies [13-15,18]. Pooled random effect analysis showed a 0.045 increase of absolute risk with urgent surgery (95% CI [-0.018; 0.109], p = 0.48). Heterogeneity assessment was conducted (Q = 2.47, p = 0.48, I^2^ = 19.7) (Figure 5).

*Summary of Results*

The pooled results for post-operative mortality, infection and re-operation rates were summarised (Table 3).

**Table 3:** Summary of Results Shows pooled absolute risk difference for mortality, infection and re-operation with associated 95% confidence intervals (CI) and P values.

Outcome	Pooled Absolute Risk Difference	95% CI	P value
Mortality	0.048	(0.027, 0.069)	p < 0.001
Infection	0.13	(-0.01, 0.27)	p = 0.20
Re-operation	0.045	(-0.018, 0.109)	p = 0.48

Sensitivity Analysis

**Figure 6:**
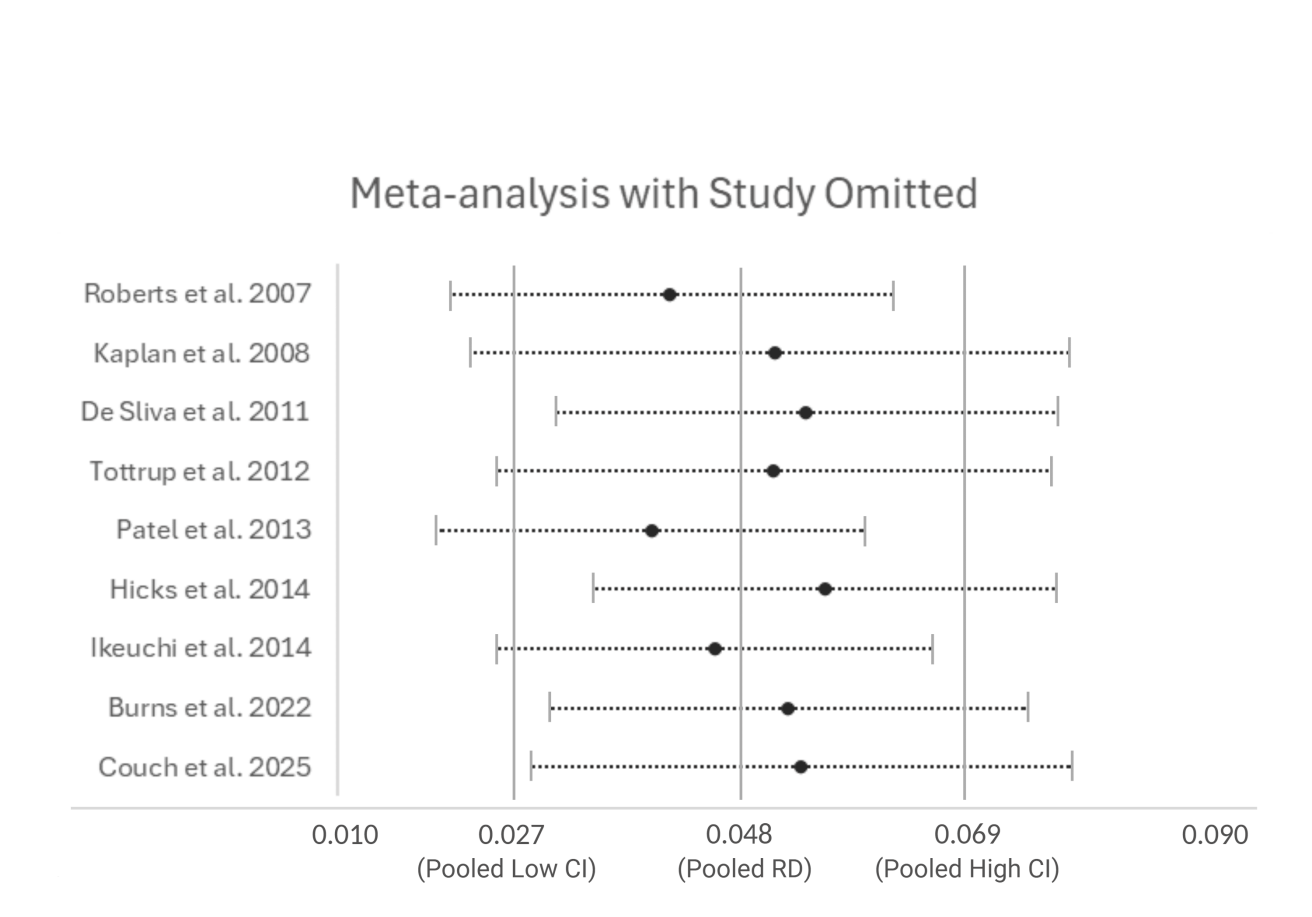
Sensitivity Analysis - Meta-analysis with Study Omitted Meta-analysis results for post-operative mortality with 95% confidence intervals when each study has been omitted in turn. Vertical lines show data from pooled meta-analysis for comparison.

Sensitivity analysis was conducted by omitting each study in turn and running the meta-analysis for post-operative mortality (Figure 6). 

Discussion

Interpretation

This systematic review and meta-analysis offers an insight into the variance in outcomes following elective versus urgent surgery for ulcerative colitis.

Post-operative mortality rates found an overall risk difference of 0.048, suggesting urgent surgery has a 4.8% increased risk of mortality compared to elective surgery. The confidence interval did not cross zero so this was statistically significant however the results showed a significant inter-study heterogeneity (Figure 3). This is consistent with mounting evidence showing patients in the acute setting are likely to be more de-conditioned and more co-morbid than their elective counterparts [20]. This difference in pre-operative morbidity between the two groups may also account for the heterogeneity within the results. Sensitivity analysis showed some variability when each study was omitted however does not account for the significant heterogeneity within the data. This heterogeneity may be due to variables present between the urgent and elective patient groups across all studies. Subgroup analysis was therefore attempted to account for pre-operative ASA grading and BMI as confounding variables. However, this raw data was not available within the majority of studies included within this review so a reliable analysis could not be performed.

Interestingly, there was no statistically significant difference in rates of post-operative infection and return to the operating theatre. Post-operative infection rates were outlined in two of the studies, with an overall risk difference of 13%; however, the confidence interval crossed zero so this was not statistically significant [15,16]. There was a 4.5% increased risk of re-operation with urgent surgery compared to elective surgery, however the confidence interval crossed zero so this was not statistically significant [13-15,18]. This is likely multi-factorial. Patients are nowadays more likely to be assessed by a wide multi-disciplinary team, allowing for more accurate timing of surgery and pre-operative planning. With improved medical treatment options for UC, there is more scope for prehabilitation, even in the acute setting. This could allow for more input from dieticians and physiotherapists to improve conditioning, as well as greater planning between surgeons and gastroenterologists.

The evolution of surgical management over time could also be responsible for the lack of difference in infection and re-operation rates. In fact, one of the studies included within this review by Couch et al. (2025) highlighted the improvement in post-operative outcomes over time [19]. This improvement is likely to be reflected in both acute and elective settings; however, there was perhaps greater room for improvement within the acute cohort. Implementation of findings from the National Confidential Enquiry into Patient Outcome and Death (NCEPOD) report (2011) and initiatives such as the National Emergency Laparotomy Audit (NELA) have significantly improved outcomes following urgent surgery over the last two decades [21,22]. These temporal trends may also account for the heterogeneity in results within this meta-analysis. Whilst only studies published within the last 20 years were included within this review, these studies often included retrospective data over several decades which is a significant confounding factor.

Clinical Implications

Whilst the advent of new immune modulators has led to more patients with medically controlled inflammatory bowel disease, surgery remains an integral part of the treatment paradigm for UC. Despite advances in surgical techniques, operative management can be challenging for patients with UC, especially in the urgent setting.

The results from this meta-analysis have implications for the way patients are counselled about the risks of surgery, both in the acute and elective setting. Patients undergoing elective surgery for UC can be counselled that deferring bowel resection could result in acute severe UC, potentially requiring urgent surgery. This would have a 4.8% greater risk of mortality. The results from this study also highlight potential scope for systemic improvement. Perhaps the use of prehabilitation and multi-disciplinary team input, even in the acute setting, may reduce the disparity in mortality rates between acute and elective UC patients.

Limitations

This study has several limitations. Given that all the studies that were used for the meta-analysis were retrospective cohort studies, this allows for selection bias. This could not be overcome within the scope of this study as a randomised-controlled trial cannot be performed to compare elective and urgent patient cohorts. Overall, studies were assessed for bias using the Newcastle-Ottawa Scale. The study by Ikeuchi et al. showed moderate risk of bias (seven stars) whilst all other studies showed low risk of bias (eight stars or more) [11-19]. Publication bias was visually assessed with the funnel plot and Egger's test was conducted. This showed no significant publication bias across the included studies. 

Secondly, several studies included retrospective data from 30 years ago, which allows for significant heterogeneity in outcomes. However, excluding these studies would reduce the volume of data and diminish the significance of the results from this meta-analysis. More contemporary cohort studies investigating post-operative outcomes in UC are therefore required to provide up-to-date and more precise results. Thirdly, the definition of urgent surgery was variable between studies with the majority of studies leaving the timing of urgent surgery undefined. Evidence suggests the timing of surgery in acute episodes of ulcerative colitis can influence post-operative outcomes [23]. Therefore, this introduces further heterogeneity within the data. Fourthly, there was significant variance between the reporting of outcomes across the studies included within the systematic review. Whilst mortality and re-operation data were often reported as 30 days post-procedure, infection rates were reported by several studies without a clear time frame. The use of an established scale, such as the Clavien-Dindo morbidity scale, across all the studies would have reduced any associated confounding variables [8]. Finally, the intervention that was investigated across all the studies was standardised by including any surgery with bowel resection. However, this fails to account for the intrinsic differences in outcome with different forms of surgery, such as with laparoscopic compared to open surgery [24]. Patients undergoing urgent surgery are more likely to have a temporary or permanent stoma whilst patients in the elective setting often undergo primary anastomosis. While a multivariate analysis was conducted, these variables could have added heterogeneity to the overall data set.

## Conclusions

Ultimately, the results from this study provide a reference frame for clinicians when managing patients with UC. In the elective setting, it can allow patients to contextualise the risks of elective surgery against potential acute flare-ups that could lead to urgent colectomies. Patients can be counselled with the increased risk of post-operative mortality when surgery is unplanned and there is physiological de-conditioning. For surgeons, this data also shows the strides that have been made to improve outcomes following urgent surgery, and highlights that further work is needed.

## Appendices

The search code used for systematic literature review was: (("colitis, ulcerative" (MeSH Major Topic)) OR ("ulcerative colitis"(Text Word)) OR ("uc"(Text Word))) AND (("elective surgical procedures"(MeSH Major Topic)) OR ("elective surgery"(Text Word)) OR ("elective resection"(Text Word)) OR ("elective colectomy"(Text Word)) OR ("elective hemicolectomy"(Text Word)) OR ("planned surgery"(Text Word)) OR ("planned resection"(Text Word)) OR ("planned colectomy"(Text Word))) AND (("emergency surgery"(Text Word)) OR ("emergency resection"(Text Word)) OR ("emergency colectomy"(Text Word)) OR ("emergency hemicolectomy"(Text Word)) OR ("urgent surgery"(Text Word)) OR ("urgent resection"(Text Word)) OR ("urgent colectomy"(Text Word)) OR ("acute surgery"(Text Word)) OR ("acute resection"(Text Word)) OR ("acute colectomy"(Text Word))) AND ((postoperative outcome(Text Word)) OR ("surgical outcome"(Text Word)) OR ("postoperative complication"(Text Word)) OR ("morbidity"(Text Word)) OR ("mortality"(Text Word))) NOT (("child"(Title)) OR ("paediatric"(Title)) OR ("infant"(Title)) OR ("neonate"(Title))) NOT (Crohn(Title)).
